# Social self-value intervention for empowerment of HIV infected people using antiretroviral treatment: a randomized controlled trial

**DOI:** 10.1186/s12879-016-1634-8

**Published:** 2016-06-10

**Authors:** Dharma Nand Bhatta, Tippawan Liabsuetrakul

**Affiliations:** Department of Public Health, Pokhara University, Nobel College, Kathmandu, Nepal; Faculty of Medicine, Epidemiology Unit, Prince of Songkla University, Songkhla, Thailand

**Keywords:** Adherence, ART, Disclosure, Self-esteem, Unprotected sexual intercourse

## Abstract

**Background:**

Prevention and antiretroviral therapy (ART) management for human immunodeficiency virus (HIV) infected people need to have long-term health care. An empowerment focused intervention is a procedure by which HIV infected people obtain combined possession of programs to attain mainly cost-effective HIV outcomes and deal with social and structural difficulties related to their universal health access and human rights. Empowerment is a key approach for addressing HIV related issues that focuses on addressing a broader context. However, the practices of empowerment based approaches are sparse. We assessed the effect of an intervention to empower HIV infected people receiving ART.

**Methods:**

In this open-label randomized controlled trial, HIV infected people from Nepal who were using ART from 6 to 24 months and were aged 18 years and above were randomly assigned to receive either the intervention or routine care. The intervention was led by two counselors for a period lasting six weeks. Participants were followed up at three and six months after the baseline. The primary outcome was change in empowerment scores, analyzed by using Difference-in-Difference (DiD).

**Results:**

Between September and November 2014, 1447 HIV infected people were screened, of whom 132 were randomly assigned to the intervention (*n* = 66) or control (*n* = 66) group. All the participants completed the 3- and 6- months follow up. A significant difference in mean empowerment score was found between the groups at 3- (46.77, *p*-value <0.001) and 6- (49.71, *p*-value <0.001) months follow up. The average treatment effect (after matching intervention and control individuals) showed that the participants who received the intervention increased their mean empowerment scores from baseline by 47.05 (*p*-value <0.001, at three months) and 49.87 (*p*-value <0.001, at six months) than those who did not receive the intervention. No adverse events were reported.

**Conclusion:**

Social self-value intervention provided to HIV infected people during ART increased their empowerment. This intervention can be expanded to be utilized in routine services.

**Trial registration:**

Thai Clinical Trials Registry, number TCTR20140814002.

## Background

Morbidity and mortality due to human immunodeficiency virus (HIV) infections can be reduced by preventing new HIV infections [1–3]. Globally, an estimated two million people were newly HIV infected in 2014 [4]. Viral load status of HIV in HIV positive people and risky sexual behavior with HIV uninfected people is the first and foremost way of transmission of HIV [5]. HIV transmission could be potentially reduced through interventions given at every step of the HIV care continuum including an efficient diagnosis system, adherence to medical care, suppression of viral load, and quality of antiretroviral treatment [6, 7]. Continuous intervention of medical care, treatment, counseling and screening might help to reduce HIV transmissions [8–10]. Persons who are HIV positive but ignorant of their HIV status have more risk behaviors of HIV transmission than those who were conscious of their infection [11, 12]. Reduction of HIV transmission is associated with antiretroviral treatment (ART) and maintaining a high retention rate in medical treatment is essential to gaining access to ART and suppression of HIV viral load [8, 9, 13].

Universal access to treatment for those who need ART is very low [4, 14]. In addition, antiretroviral drug toxicity, resistance and non infectious developments are the major challenges for maintaining a higher retention rate in regular medical care [15–17]. Furthermore, low negotiation skills of risky sexual behavior with sexual partners, social problems, co-infections and re-infections make HIV infected people more vulnerable [18, 19]. Existing studies of HIV interventions and non-interventions have focused on primary and secondary prevention of subgroups and clinical implications for mortality and morbidity rather than the possible outcome for HIV prevention among all HIV infected people [6, 7, 20–24]. Moreover, overambitious targets related to HIV including zero new infections, discrimination and stigma have been established by various agencies [25–27]. However, gold standard interventions have been unable to address those targets. An empowerment approach would be an asset to achieve above HIV related inequalities and targets [28–31]. While health outcomes and self care behaviors are linked to empowerment methods with a supportive social environment, it must be considered for implementation in programs and policies to increase epidemiological profits on investment [32, 33].

An empowerment approach has not yet been developed and tested for all HIV infected people. Empowerment is a cost-effective approach to reduce HIV transmission, improve treatment retention, and reduce social, physical and psychological problems [34, 35]. This social self-value intervention package was developed on the basis of the diffusion model of innovations study [36]. The concept of social action and empowerment theories were used to enhance self-efficacy, self-care, family and social relationships, stigma and discrimination issues [37–41] which are the major obstacles among HIV populations. As a result, self-esteem, autonomy, social adaptation or relationship and behavior change for structural prevention as an empowerment framework could be strengthened [20–24, 42, 43]. We aimed to assess the effect of this intervention on empowerment of HIV infected people receiving ART.

## Methods

### Study design, settings and participants

**Fig. 1:**
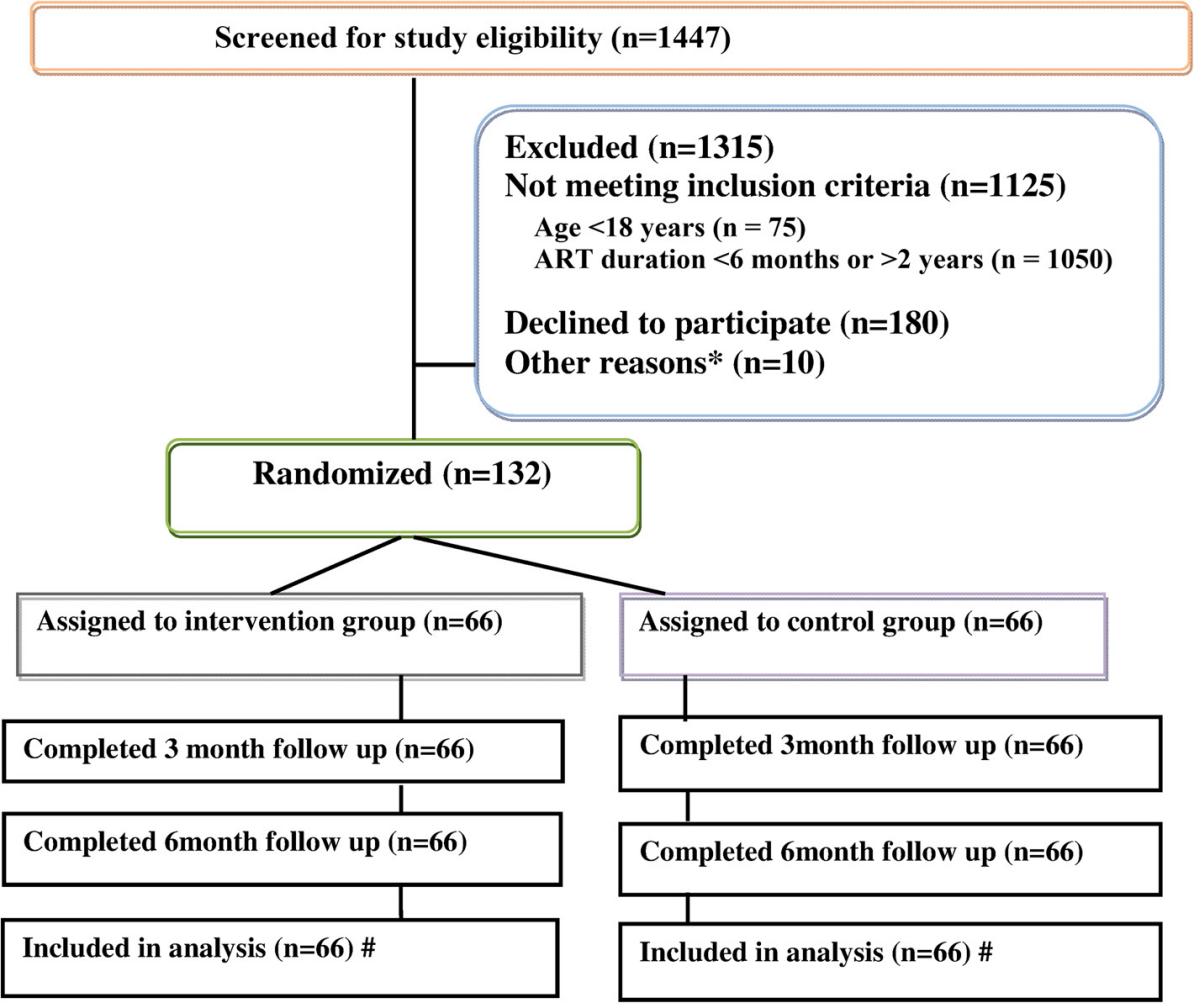
Study design and participant enrolment flow diagram. Legend: *Patients transferred out or did not come to the center during recruitment period. #intention-to-treat design

This open label, parallel, randomized controlled trial was conducted in Kathmandu, Nepal. HIV infected participants receiving antiretroviral treatment (ART) from ART centers of Kathmandu district of Nepal were recruited. The study was conducted from September 2014 to June 2015. The study site was Sukraraj Tropical and Infectious Disease Hospital (STIDH), Teku, Kathmandu run by the government of Nepal and was a part of the National Center for AIDS and STD Control (NCASC) [44]. This site is the largest ART center catering to both rural and urban people living in Nepal. It has provided multidisciplinary clinical and laboratory services and treatment for HIV infected people since 2004 [45]. Figure 1 shows details of the study design and participant enrollment.

Eligible participants included HIV infected people aged 18 years or above, willing to participate in either intervention or control arm, and had been receiving ART between 6 and 24 months prior to the study according to the ART criteria as per the guidelines of NCASC [46]. We excluded participants who were exposed to similar educational programs or any other intervention, expressed inability to attend all the study follow up periods, suffering from health problems (psychotic disorders, visual and hearing problems), and unwilling to disclose their HIV status among other participants.

A total sample size of 132 participants (66 in the control arm and 66 in the intervention arm) would achieve 80 % power to detect a significant mean difference of empowerment scores at a level of 0.05. Since, no previously published study was found for the mean change of intervention (μ_1_ -μ_2_), a 20 % mean difference was applied which was equal to 0.52 standard deviation (σ). The formula for testing the difference between two means (two-sample *t*-test) was used to arrive at the sample size.

### Randomization and masking

Participants were randomly allocated to either the intervention or control arm with a ratio of 1:1. Randomization was performed by a random number generator with permuted blocks of six. Allocation concealment was done by using sequentially numbered opaque sealed envelopes. The independent data manager generated the randomly sequence numbers. The sequence numbers were masked from other research staff and participants. None of the research team members and participants was involved in the randomization process and subsequent to randomization none of the participants were able to modify their assignment. Enumerator and analysis assessor were masked from baseline to follow-up data by using a unique code. The unique code was developed for all the participants by the team leader to maintain the anonymity of the participants.

### Intervention procedures

Many theories were followed to develop the intervention package. The intervention contents were developed based on social learning, social action, and pedagogy theory and empowerment principles for HIV prevention and treatment [20–24, 37–43].

All the participants completed a baseline survey before the intervention began. Once participants were recruited and assigned their allocation baseline characteristics were collected.

All participants who met the selection criteria were informed about the study process, design and goals. All subjects in the intervention group attended six intervention sessions of one and half hours duration at the ART center. Intervention sessions were conducted once per week and per session eight to ten participants were included. All the sessions were led by two national level trainers with public health degrees. The intervention sessions were as follows: first session covered rapport building, sharing uncomfortable situations and management of negativity; second session started with barriers and strategies of HIV disclosure and defeat with stigma and self-esteem; third session covered healthy body and healthy mind, healthy sexual relations, means to be HIV infected or non-infected to be a man or woman, sexuality, adherence of ART and other treatment and prevention strategies after infection; fourth session started with strategies to plan for healthy relations with family members, the community and society, effective communication, and responsibilities in the society; fifth session discussed negative effects of illicit drugs, alcohol, and smoking, skills for co-infection, re-infection and partner’s sexual behavior, diet and exercise; sixth session educated about legal empowerment, human rights, legal protection, discrimination, stress, rising voice together against discrimination and rights and future goals.

Acceptability, applicability and relevancy of the intervention contents were discussed with two experts, three HIV infected people and two counselors. After necessary amendment, the contents for all six sessions were pre-tested among ten HIV infected people. Participatory learning activities, buzz sessions, brain storming, lecturers, and discussion techniques were used in the intervention sessions. Participants were instructed to discuss the issues within their group and other participants. Participants were encouraged to communicate with their family members, peer groups, friends related to HIV control, prevention, treatment and disclosure. Participants were assigned home assignments at the end of each session for presentation at the next session. All the participants in the intervention group were compensated for each of their six sessions with an amount equivalent to US $ 20 in the local currency. The control group did not receive any compensation.

Fidelity of the intervention was maintained with continuous monitoring of the allocated time for topics, methods and contents of the sessions by health officers and the research team leader. In addition, anonymity was maintained with a code and participants were assured quality during the discussion sessions. To maintain compliance, at the end of the each session the counselors motivated participants to participate in the next session, encouraged voluntary independent participation and provided gift vouchers. The overall participant retention rate was 96.6 % in the intervention session.

### Standard care

All participants received routine standard care as per the NCASC guidelines [46]. This included pre ART counseling, routine medical and laboratory tests and monthly follow up for ART. Standard care in Nepal is provided by government organizations and ART is dispensed free of cost.

### Study procedures

Participants were asked to provide information on demographics, empowerment and behaviors at baseline and follow up (first follow up: 3 months after the baseline and second follow up: 3 months after the first follow up). To minimize the errors and enhance quality control, double data entry was employed and extensively supervised by the research team leader. Anonymity and confidentiality were maintained with assigned unique codes. Intervention contents and tools were pre-tested before baseline and follow up data collection.

### Measures

Demographic characteristics included age, sex, ethnicity, religion, occupation, education, marital status, children and per capita family income.

The primary outcome was measured by using an empowerment scale developed by Rogers et al. [47], containing a total of 28 items each measured on a four-point agreement scale ranging from strongly disagree to strongly agree. Total empowerment scores ranged from 28 to 112 consisting of five subscales, namely self-efficacy/self-esteem (9-36 score), power–powerlessness (7-28 score), community activism and autonomy (5-20 score), optimism and control over the future (4-16 score) and righteous anger (3-12 score). First, the contents of the questionnaire were discussed with two experts who amended the language for suitability with HIV patients. Further contents were revised to be applicable to the Nepalese culture and contexts. The content was then discussed with three HIV infected people for clarity and acceptance, and amended accordingly. After development of the revised version with experts and HIV infected people, the final version was pretested among HIV infected people. During the pre-test, no negative comments or difficulties were encountered by participants. Internal consistency, as measured by Cronbach’s alpha was 0.97.

 disclosure of HIV status (coded as yes or no, if response was yes then the number of persons disclosed was recorded and dichotomized as ≤3 persons or >3 persons). These secondary outcomes were measured to assess the effect of empowerment to patient’s behaviors which were important to their health outcomes.

### Statistical analyses

Demographic characteristics were compared between the intervention and control group at baseline. Baseline differences between the two groups were tested using Fisher’s exact or Chi-squared test for categorical variables and unpaired t-tests or Wilcoxon’s signed rank test for continuous variables as appropriate.

Analysis of the primary outcome emphasized the differences of empowerment scores among HIV infected people comparing between the intervention and control groups at baseline (baseline difference or pre-difference) and at 3- (post-difference at 3 months) and 6- month post-intervention (post-difference at 6 months). The impact of the intervention on empowerment was analyzed by comparing Difference-in-Differences (DiD) scores. The impact of the intervention was measured between baseline difference and post difference at 3 months (DiD_3mo_) and between baseline difference and post difference at 6 months (DiD_6mo_). The secondary outcomes were analyzed at baseline, 3- and 6- months follow up using univariate analysis. Significant differences among intervention and control were measure using Fisher’s exact test.

#### Difference in Differences (DiD)

DiD methods can be used to estimate causal relationships [48]. DiD compare the differences in outcomes among the intervention group in pre- and post-intervention and involves indentifying similar differences among the control group. We used DiD to compare outcomes between control and intervention groups at baseline, 3- and 6- months follow up [49]. Ordinary Least Square (OLS) with repeated data for control and intervention group for baseline, 3- and 6- month follow up periods produced standard errors and DiD estimates. The equation considered as follows:

$$ {\mathrm{Y}}_{\mathrm{ist}}={\mathrm{A}}_{\mathrm{s}} + {\mathrm{B}}_{\mathrm{t}} + \mathrm{c}{\mathrm{X}}_{\mathrm{ist}} + \upbeta {\mathrm{I}}_{\mathrm{s}\mathrm{t}}+{\mathrm{e}}_{\mathrm{ist}} $$

where empowerment is the outcome of interest, denoted as*Y*_*ist*_ for the individual of HIV infected *i* in randomized group *s* (control or intervention) by time *t* (the baseline and 3- or 6- months follow up) and *I*_*st*_ is an indicator variable representing whether the intervention has affected the group *s* at time *t. A*_*s*_ and *B*_*t*_ are fixed effects for the randomized group and time (baseline and follow up) respectively, *X*_*ist*_ are applicable individual controls and e_ist_ is the error term. The impact of the intervention was estimated by OLS with *^*β estimation. Means and standard errors were estimated by linear regression. Following linear regression for the individual *i*:

$$ \mathrm{Outcom}{\mathrm{e}}_{\mathrm{i}} = {\upbeta}_{\mathrm{o}} + {\upbeta}_1 \times \mathrm{perio}{\mathrm{d}}_{\mathrm{i}} + {\upbeta}_2 \times \mathrm{treate}{\mathrm{d}}_{\mathrm{i}}+{\upbeta}_3 \times \mathrm{perio}{\mathrm{d}}_{\mathrm{i}} \times \mathrm{treate}{\mathrm{d}}_{\mathrm{i}} + {\mathrm{e}}_{\mathrm{i}} $$

where, ^^^β_3_: is the DiD or impact.

The analyses were done with R and Stata (*diff* command) was used for estimating the DiD [50]. All tests were two sided and a *p*-value of <0.05 indicated statistical significance.

### Ethical considerations

Comprehensive privacy was maintained during the study period. Strict anonymity and confidentiality was maintained throughout the recruitment, intervention and data collection process by using unique codes. A standard protocol was followed to maintain data safety and confidentiality of the study data [51]. In agreement with national guidelines and the principles of the declarations of Helsinki, written informed consent was obtained from all participants prior to enrollment. The right to withdraw at any time and skip any question was offered to all participants. We provided travel costs and reimbursement during the intervention and follow up periods. Researchers had no direct or financial benefits and declared no conflict of interest.

This project was approved by the Research Ethics Committee, Faculty of Medicine, Prince of Songkla University, Thailand (reference no. 57-0146-18-5) and approved by Institutional Ethical Review Committee of Sukraraj Tropical and Infectious Disease Hospital (STIDH), Nepal (063/071/72) prior to study initiation. The trial was registered through trial registration number TCTR20140814002 (www.clinicaltrials.in.th). All the contents were reported as per the CONSORT guidelines.

## Results

A detail of the trial profile is shown in Fig. 1. One hundred thirty-two participants were randomly assigned to receive the intervention (*n* = 66) or no intervention (*n* = 66) between September and November 2014. 1447 individuals were screened and of these, 1125 were ineligible, 180 declined to participate and 10 were excluded due to being transferred out or because they did not come to the center during the recruitment period. All participants in both groups were retained in the study at three and six months follow-up (100 % retention rate).

No significant differences at baseline were observed in demographic characteristics of the participants between the intervention and control groups. The mean ages of participants in the intervention and control groups were 36.3 and 35.8 years, respectively. The majority had a low family income, was married and had children (Table 1).

**Table 1:** Baseline demographic characteristics

	Control group	Intervention group	*p*-value
	(*n* = 66)	(*n* = 66)	
Age (years)			
Mean(SD)	35.8 (8.8)	36.3 (6.8)	0.71
≤36	36 (54.5)	41 (62.1)	0.48
>36	30 (45.5)	25 (37.9)	
Gender			0.22
Female	39 (59.1)	31 (47)	
Male	27 (40.9)	35 (53)	
Ethnicity			0.60
Indigenous	31 (47.0)	27 (40.9)	
Non-indigenous	35 (53.0)	39 (59.1)	
Religion			0.44
Hindu	49 (74.2)	44 (66.7)	
Others	17 (25.8)	22 (33.3)	
Occupation			0.85
Unemployed	22 (33.3)	21 (31.8)	
Informal employee	25 (37.9)	23 (34.8)	
Formal employee	19 (28.8)	22 (33.3)	
Education level			0.10
Illiterate informal	29 (43.9)	19 (28.8)	
Primary and above	37 (56.1)	47 (71.2)	
Marital status			0.10
Single	17 (25.8)	17 (25.8)	
Married	49 (74.2)	49 (74.2)	
Children			0.09
No	14 (21.2)	6 (9.1)	
Yes	52 (78.8)	60 (90.9)	
Number of children			0.08
≤2	39 (59.1)	50 (75.8)	
>2	13 (19.7)	10 (15.2)	
Family per-capita income (USD^a^)			
Median(IQR)	50 (30.67)	50 (30.67)	0.81
≤50	41 (62.1)	44 (66.7)	0.72
>50	25 (37.9)	22 (33.3)	

^a^ *1USD* 100NPR, *IQR* inter quartile range, *SD* standard deviation

**Fig. 2:**
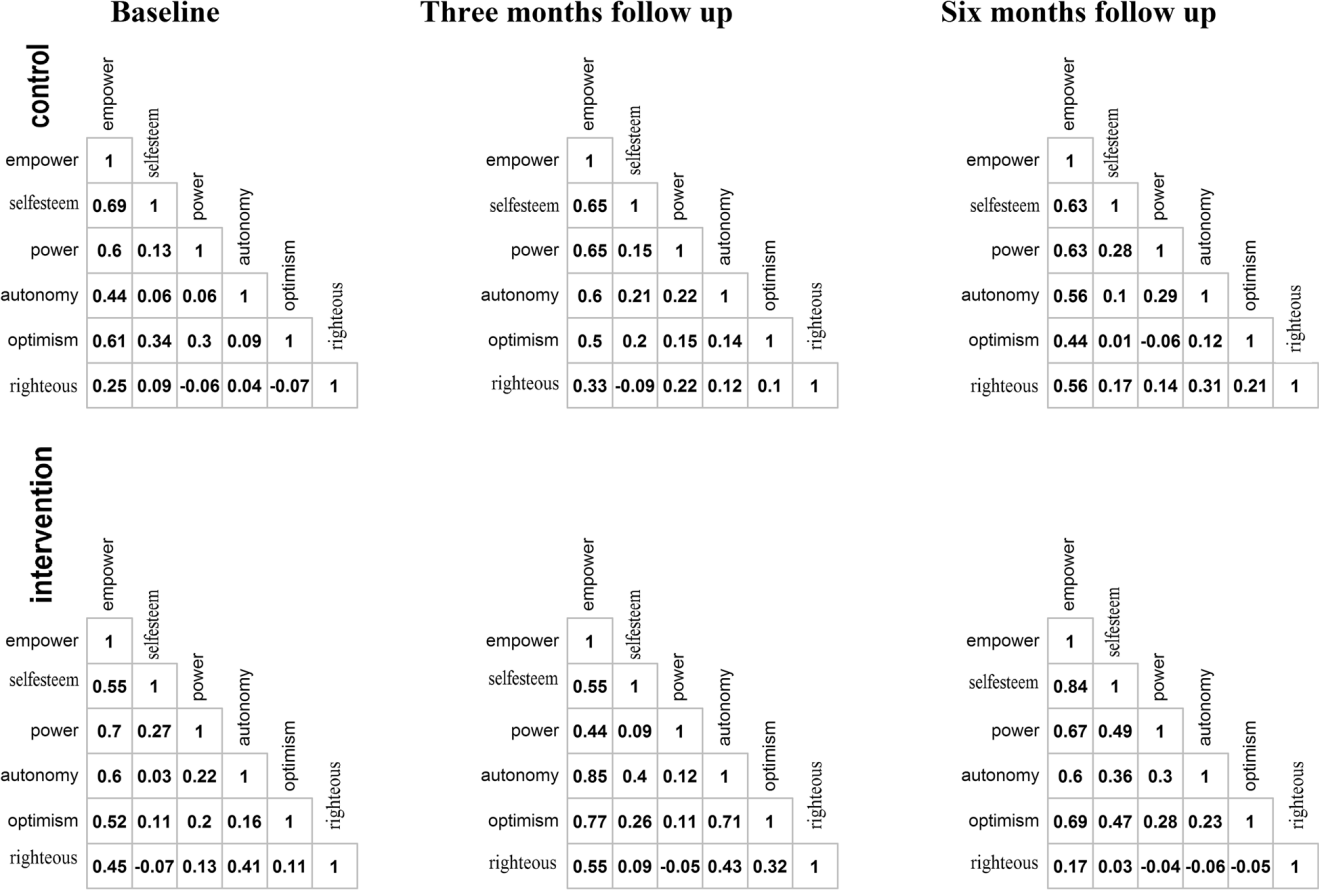
Correlation among domains and total empowerment score. Legend: empower: empowerment total score; selfesteem: self-esteem/self-efficacy; power: power-powerlessness; autonomy: community activism and autonomy; optimism: optimism and control over the future; righteous: righteous anger

The correlations among empowerment domains and total empowerment score are presented in Fig. 2. Total scores of empowerment were positively correlated with the other five domains at baseline, 3- and 6- months follow up. In the intervention group at the 6 months follow up, power-powerlessness, community activism and autonomy, optimism and control over the future were negatively correlated with righteous anger, and were lowly correlated with self-efficacy/self-esteem and righteous anger.

The impact of the intervention on empowerment is presented in Table 2. The baseline difference (pre-difference) of empowerment scores between the intervention and control groups were not significantly different. Difference-in-Difference at 3- (46.77, *p* <0.001) and 6- months (49.71, *p* <0.001) were significantly higher for the intervention group in all domains of empowerment.

**Table 2:** Impact of intervention on empowerment

	Baseline		3 month follow up		Pre-Diff	Post-Diff_3mo_	DiD_3mo*_ (Impact)	6 month follow up		Post-Diff_6mo_	DiD_6mo*_ (Impact)
	Control	Intervention	Control	Intervention				Control	Intervention		
Empowerment (total score)	46.70	46.38	48.23	94.68	−0.32	46.45	46.77	46.53	95.92	49.39	49.71
Self-efficacy/self-esteem	15.27	15.03	15.39	30.33	−0.24	14.94	15.18	15.12	30.91	14.79	16.03
Power-powerlessness	12.00	12.03	12.26	23.92	0.03	11.67	11.64	11.67	24.18	12.51	12.48
Community activism and autonomy	8.01	8.04	8.65	16.83	0.03	8.18	8.15	8.26	16.92	8.67	8.64
Optimism and control over the future	6.59	6.50	6.61	13.54	−0.09	6.94	7.03	6.36	13.41	7.04	7.14
Righteous anger	4.82	4.77	5.32	10.04	−0.04	4.73	4.77	5.12	10.50	5.38	5.42

* = *p* <0.001; *Pre-diff* difference at baseline, *Post-diff*_*3mo*_ difference at 3 month follow up, *Post-diff*_*6mo*_ difference at 6 month follow up, *DiD*_*3mo*_ difference at baseline and 3 month follow up, *DiD*_*6mo*_ difference at baseline and 6 month follow up

After standardization of scores for each domain, the impact of the intervention on each domain of empowerment remained equal. Participants who received the intervention increased their empowerment scores by an average of 47.05 points (*p*-value < 0.001, DiD at three months and baseline) and 49.87 points (*p*-value < 0.001, DiD at six months and baseline) more than those who did not receive the intervention, after propensity score matching of intervention and control individuals (data not shown).

**Fig. 3:**
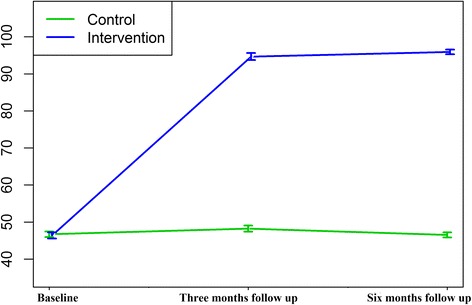
Trend of average empowerment score with 95 % confidence interval for intervention and control groups

Figure 3 depicts the trend of average empowerment scores among the two groups at baseline, 3- and 6- months follow up. The mean post-intervention score of empowerment markedly increased among the intervention group at 3 months but only slightly increased at 6 months, while the mean score for the control group remained constant.

Table 3 shows pre- and post-intervention differences on behavioral and clinical characteristics. From baseline to 6 months, unprotected sexual intercourse with any partner decreased in the intervention group and increased in the control group. The proportion of those who ever forgot to take ART did not change in the intervention group but the proportion rose from baseline to 6 months among the control group. No participant forgot to take ART in the past week in the intervention group. The proportion of participants who disclosed their HIV status rose from baseline to 6 months in the intervention group. Focusing on change from baseline to 6 months, statistically significant differences between the control and intervention groups were found for unprotected sexual intercourse (*p*-value <0.001), forgetting to ever take ART (*p*-value 0.007), forgetting to take ART in the past week (*p*-value <0.001), and disclosure of HIV status (*p*-value <0.001).

**Table 3:** Pre- and post-intervention differences on behavioral and clinical characteristics

	Baseline			Three month follow up			Six month follow up		
	Control	Intervention	*P*-value	Control	Intervention	*P*-value	Control	Intervention	*P*-value
	(*n* = 66)	(*n* = 66)		(*n* = 66)	(*n* = 66)		(*n* = 66)	(*n* = 66)	
Unprotected sexual intercourse with any partner in last 3 months^a^			0.82			0.001			0.001
No	38 (76.0)	36 (72.0)		19 (35.8)	47 (100.0)		19 (36.5)	45 (95.7)	
Yes	12 (24.0)	14 (28.0)		34 (64.2)	0 (0)		33 (63.5)	2 (4.3)	
Ever forgot to take ART			0.16			0.38			0.007
Yes	23 (34.8)	32 (48.5)		26 (39.4)	32 (48.5)		48 (72.7)	32 (48.5)	
No	43 (65.2)	34 (51.5)		40 (60.6)	34 (51.5)		18 (27.3)	34 (51.5)	
Forgot to take ART in past week			0.78			0.001			0.001
Yes	6 (9.1)	8 (12.1)		56 (84.8)	0 (0)		20 (30.3)	0 (0)	
No	60 (90.9)	58 (87.9)		10 (15.2)	66 (100.0)		46 (69.7)	66 (100.0)	
Disclosure of HIV status with^b^			0.93			0.001			0.001
≤ 3 persons	46 (80.7)	45 (77.6)		46 (76.7)	18 (27.3)		44 (73.3)	2 (3.0)	
> 3 persons	11 (19.3)	13 (22.4)		14 (23.3)	48 (72.7)		16 (26.7)	64 (97.0)	

^a^Missing data ^b^among disclosed, *p*-value = Fisher’s exact test

## Discussion

A social self-value intervention package was shown to empower HIV infected people receiving ART and improved their behaviors. Our study highlighted a significantly greater increase in empowerment for HIV infected people at 3- and 6- months from the baseline. Similar findings were highlighted by a quasi-experimental study from Canada that anticipated empowering HIV infected people [52]. However, there are sparse existing studies available related to empowerment of all HIV infected people [52, 53].

Baseline characteristics of the participants were not statistically significant different among the intervention and control groups. Further we collected background characteristics (age, sex, ethnicity, marital status, date ART started) for all the screened participants. There was no difference in these background characteristics between those who agreed to participate in the study and those who refused thus minimizing biological, environmental and socioeconomic bias. The given reasons for refusal to participate were lack of interest, time and perceived need for the intervention. The strong recruitment process enhanced a higher retention rate in the intervention group and lower loss to follow up at 3- and 6- months. The intervention sessions took place in the same center where the controls received ART. This could have increased the chance of contamination among the control group. To reduce this risk, we conducted the intervention after services had finished for the day in each center and participants were counseled not to disclose any activities during the study period. The fact that the findings did not show any changes among the control group after the intervention provides evidence of no or minimal contamination. On the other hand, provided incentives to the intervention group could lead to confirmation bias. Incentivized group might be more biased in their information than who did not get incentives.

The total empowerment score was highly correlated with its different domains but different domains were loaded with various correlations from high (self-efficacy) to low (righteous anger). Self-efficacy/self-esteem domain revealed a significant enhancement at six months which was similar to findings from previous studies using different interventions for HIV infected populations [52, 54, 55]. It was confirmed in a systematic review on community-based interventions that empowerment intervention had positive effects on self-esteem [56]. Self-esteem is necessary to enhance the management of the negative social, physical and emotional impacts of HIV infection, thus our intervention was useful to support these issues.

Righteous anger domain was negatively correlated with the other domains. This might be the effect of the intervention that operates to reduce feelings of revenge over perceived mistreatment among HIV infected people. In addition, greatly increased righteous anger (reactive feeling of fury over abuse) scores had a positive influence on social and community adaption and adjustment of HIV infected people. Our trial showed a significantly greater increase in community activism and autonomy, power-powerlessness (helpless and totally incompetent) and optimism and control over the future (hope and assurance about the future or successful result of something). This may have affected the capacity to rebalance and reincorporate their lives [57]. HIV infection might guide the person to the destruction of their life goals, as well as absence of autonomy and self-control. Powerlessness and lack of control over the future appears as a diverse risk factor of disease. The impact of the intervention was further validated by using an average treatment effect model with propensity score matching. This was done to reduce the bias, although the participants were randomly assigned and no significant changes were reported after the modeling. Findings revealed significant improvements in empowerment score from baseline to 3 months follow up, but only minimal improvements at six months. The improvements at three months might be due to the immediate effect of the intervention while lack of further improvements at 6 months is probably due to a ceiling effect – the optimum empowerment score may have already been achieved. However, this needs long-term follow up to illicit the possible effects and reasons.

Our intervention improved not only empowerment but also the behavior of HIV infected people most likely because behavior related contents were included in the intervention package. A systematic review of interventions showed both significant and non-significant positive effects of interventions to reduce risk behaviors [58]. Our study found that the practice of unprotected sexual intercourse among HIV infected people with any partner was significantly reduced after the intervention. A systematic review on community empowerment interventions for HIV prevention showed a reduction in risky sexual behaviors and increase in condom use among sex workers [59]. Another study related to empowerment of young HIV infected people showed a significant improvement in protected sexual intercourse [55]. This trial revealed a significant improvement in adherence to ART among the intervention group and a decrease among the control group. Previous interventions highlighted that the empowerment of HIV infected people showed an improvement in adherence to ART [52, 60]. Our study highlighted a significant increase in HIV status disclosure rate. Disclosure is important to prevent the spread of HIV, increase the wisdom of self-esteem, emotional and practical support from social networks [61]. An empowerment intervention was envisioned with a multi-level construct that entails an understanding of social adaptation or relationship, self-esteem, autonomy, and behavior change for structural prevention throughout the contribution of developed skills, strengths and advocacy to behavioral, social interdependence and cognitive changes. This outcome might be the path of effect that is associated with empowerment theory.

Studies related to empowerment to all the HIV infected people were not available in this region. Although applicable to local culture and context intervention package, a highly experienced interventionist and extensive quality control measure might be the reason for improvement of empowerment scores and behavioral outcomes among the intervention group. Although subgroup analysis was found significant in small sample size and we suggested evaluating in future multicenter and large sample size based intervention and long term effects. An empowerment measurement tool would yield two dimensions of self and community directions to empowerment. Community orientation to empowerment believed that HIV infected people have power inside the society and desire to encourage community action in an unfriendly world. Self orientations to empowerment believe themselves to be self-esteem, self-efficacious, and optimistic to the future.

### Strengths and limitations

This trial was based on randomly assigned participants, a blinded analysis process and use of rigorous outcome analysis guaranteeing high internal validity. The intervention package and measurement tools were pre-tested in different stages which increased its reliability. The intervention package was found acceptable and feasible after measurement by both qualitative and quantitative approaches [62]. A high retention rate in the intervention group as well as during follow up was maintained. The intervention was conducted in the regular health care service setting provided by the Nepalese government which pretend the real world setting and added to the external validity. Our study population characteristics including socio-demographic and clinical features were consistent with other HIV infected populations in Nepal. Therefore, the results can be generalizable to other HIV infected people. We verified participants ART adherence with their records in the ART center to reduce the potential desirability of reporting and recall biases. However, we did not execute the pill count measure.

There were some limitations in this study. First, participants were not blinded to the intervention. However, a rigorous coding system ensuring the anonymity was used with enumerator for data collection, entry and analysis. Second, reduced risky sexual behavior, high adherence and disclosure rate found in this study could be due to the Hawthorne effect (benefit of trial participation in the intervention group), which can eliminate the power to detect a factual difference from a trial [63]. Drug toxicity, accessibility and attitude might be potential confounders for adherence to ART. Further, these changes might have happened due to social desirability bias. However, a good rapport building during the intervention and data collection, familiar enumerator and study settings to participants all helped to reduce this bias. Third, a randomized controlled trial is a dynamic design that can reduce bias due to confounding. However, there are inbuilt biases that might be mostly pertinent in behavioral intervention trials. Factors such as process of informed consent, study measurement tools that are used many times and reimbursement for participation in the trial could add to changes in behaviors among both control and intervention groups. Fourth, this empowerment intervention did not cover economic aspects. Finally, factor analysis was not used due to sampling inadequacy. However, after face validity and few modifications to language, the reliability was tested and showed good internal consistency. In other settings when empowerment measurement tool will be applied, the validity and reliability should be evaluated before use in different local cultures and contexts.

## Conclusion

The efficacy on empowerment of HIV infected people using ART was shown after receiving the intervention. Their risky sexual behaviors were reduced and their adherence to ART and disclosure of HIV were increased. The intervention contents can be utilized in regular services and its effectiveness needs to be evaluated after routine implementation. Further, the empowerment intervention framework and method of measurement can be used in different settings after validating its cultural and contextual acceptability and applicability.

## Abbreviations

ART, antiretroviral therapy; DiD, Difference-in-Differences; HIV, human immunodeficiency virus; IQR, inter quartile range; NCASC, National Center for AIDS and STD Control; SD, standard deviation; STIDH, Sukraraj Tropical and Infectious Disease Hospital; USD, United States Dollar
